# The safety and efficacy of BCG encapsulated alginate particle (BEAP) against *M.tb* H37Rv infection in *Macaca mulatta* : A pilot study

**DOI:** 10.1038/s41598-021-82614-5

**Published:** 2021-02-04

**Authors:** Ashwani Kesarwani, Parul Sahu, Kshama Jain, Prakriti Sinha, K. Varsha Mohan, Puja S. Nagpal, Surender Singh, Rana Zaidi, Perumal Nagarajan, Pramod Upadhyay

**Affiliations:** 1 National Institute of Immunology, Aruna Asaf Ali Marg, New Delhi, 110067 India; 2 Department of Biochemistry, Jamia Hamdard, New Delhi, 110062 India

**Keywords:** Immunology, Diseases, Medical research

## Abstract

Due to the limited utility of Bacillus Calmette–Guérin (BCG), the only approved vaccine available for tuberculosis, there is a need to develop a more effective and safe vaccine. We evaluated the safety and efficacy of a dry powder aerosol (DPA) formulation of BCG encapsulated alginate particle (BEAP) and the conventional intradermal BCG immunization in infant rhesus macaques (*Macaca mulatta)*. The infant macaques were immunized intratracheally with DPA of BEAP into the lungs. Animals were monitored for their growth, behaviour, any adverse and allergic response. The protective efficacy of BEAP was estimated by the *ex-vivo* H37Rv infection method. Post-immunization with BEAP, granulocytes count, weight gain, chest radiography, levels of liver secreted enzymes, cytokines associated with inflammation like TNF and IL-6 established that BEAP is non-toxic and it does not elicit an allergic response. The T cells isolated from BEAP immunized animals’ blood, upon stimulation with *M.tb* antigen, secreted high levels of IFN-γ, TNF, IL-6 and IL-2. The activated T cells from BEAP group, when co-cultured with *M.tb* infected macrophages, eliminated largest number of infected macrophages compared to the BCG and control group. This study suggests the safety and efficacy of BEAP in Non-human primate model.

## Introduction

BCG (Bacillus Calmette–Guérin) is a live attenuated form of *Mycobacterium bovis* and it is the only vaccine available and used for tuberculosis worldwide. It has been demonstrated that the BCG vaccine renders protective immunity in infants’ till up to 15 years of age^1,2^. However, it fails to protect in areas of high TB burden regions^3^.

There are a few new vaccine candidates in the pipeline^4^ but so far it has been challenging to provide protection any better than the BCG. In many of the vaccine candidates, mycobacterium similar to BCG have been evaluated^3^. Alternatively, BCG has been modified genetically to improve its efficacy^5–8^. New formulations and delivery routes have also been investigated^9–11^.

In this context, we had previously reported a unique dry powder aerosol (DPA) formulation of BCG^12^ as a potential vaccine candidate. This formulation is a BCG encapsulated alginate particle (BEAP), which is 2–5 μm in size and to be delivered as an aerosol. The formulation remains viable for 6 months at room temperature.

It is now generally accepted that the immunization by aerosol of BCG provides better protection against *M.tb* infection than the conventional intradermal immunization^10,11^. In our previous report, we have demonstrated that the alginate coated DPA formulation of BCG, the BEAP offers enhanced protection as compared to BCG aerosol immunization in mice^12^. To advance the study towards human applicability, the next obvious step is to investigate the performance of this formulation in a primate model like the *Macaca mulatta* since these animals manifest the full spectrum of disease conditions as in humans^13^.

Hence, in this pilot investigation, the dry powder formulation, BEAP was evaluated in Indian rhesus macaques *(Macaca mulatta)*. To maintain similarity with the immunization schedule of BCG in humans, we compared the intratracheal immunization of BEAP in infants’ macaques with conventional intradermal BCG and un-immunized controls.

We assessed the safety of the BEAP formulation in infant macaques by monitoring behaviour and growth, liver function tests and cytokines level in serum for up to 12 months post immunization. The efficacy of the formulation was evaluated by an *ex-vivo* infection assay with *M.tb* H37Rv.

## Results

### Standardization of immunization procedures

**Figure 1:**
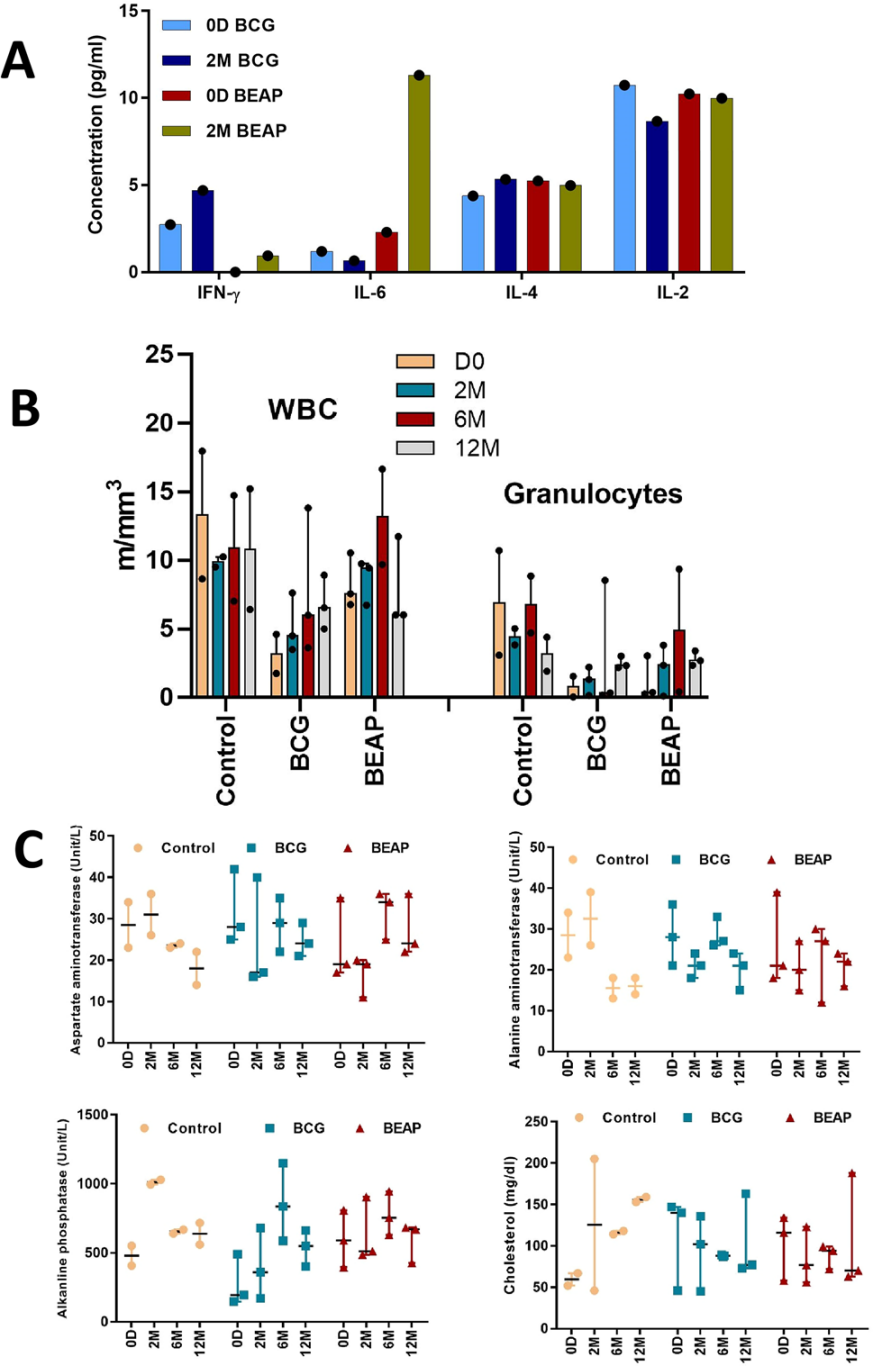
Evaluation of the toxicity or allergenicity of BEAP formulation. (**A**) Cytokine levels in the serum of BCG/BEAP immunized adult animal on D0 and at 2 months post immunization, (**B**) The granulocytes and WBC count in the control and post immunized infants. (**C**) Levels of liver secreted enzymes aspartate aminotransferase (AST), alanine aminotransferase (ALT) alkaline phosphatase (ALP) and cholesterol in the control and BCG/BEAP immunized infants at different time points. Dots indicate data points and bars are median with 95% confidence interval. Graphs were plotted using GraphPad Prism version-7 program (https://www.graphpad.com/).

The procedure of immunization in infant macaques was first evaluated in two adult macaques. One of the animals was immunized intradermally using a conventional BCG vaccine and the other was immunized intratracheally with DPA of BEAP. The intratracheal intubation and administration of DPA procedures were well tolerated by the animal. They were monitored up to 2 months post immunization during which they remained normal and healthy. Figure 1A shows levels of IFN-γ, IL-6, IL-4 and IL-2 in the serum of BCG/BEAP immunized adult animal on D0 and at 2 months post immunization. Normal levels of these cytokines in these animals confirmed no adverse reaction post immunization.

### BEAP is non-toxic and does not elicit an allergic response

The subsequent experiments were done on 8 macaque infants; 2 un-immunized controls, 3 BEAP immunized and 3 BCG immunized infants. Post-immunization all the animals were observed for any adverse effects due to the formulation. The blood and serum were checked for inflammatory or allergic response through the Complete blood count (CBC), cytokine profile and toxicity were checked through Liver Function Tests (LFT).

#### Granulocytes and WBC counts

CBC test was performed in fresh un-clotted blood of the control and BCG/BEAP immunized infants. The granulocyte and WBC count were in the normal range and suggested no adverse reactions post immunization (Fig. 1B) and Supplementary data Table 2.

#### LFT

Liver secreted enzymes, aspartate aminotransferase (AST), alanine aminotransferase (ALT), alkaline phosphatase (ALP) and cholesterol production levels were checked in the serum of the control and BCG/BEAP immunized animals using biochemical assay kits.

The LFT profile revealed that in the majority of the animals, all the enzymes and cholesterol levels were found within the normal reference range (Fig. 1C), suggesting that there was no toxicity due to immunization.

#### Cytokines in Serum

**Figure 2:**
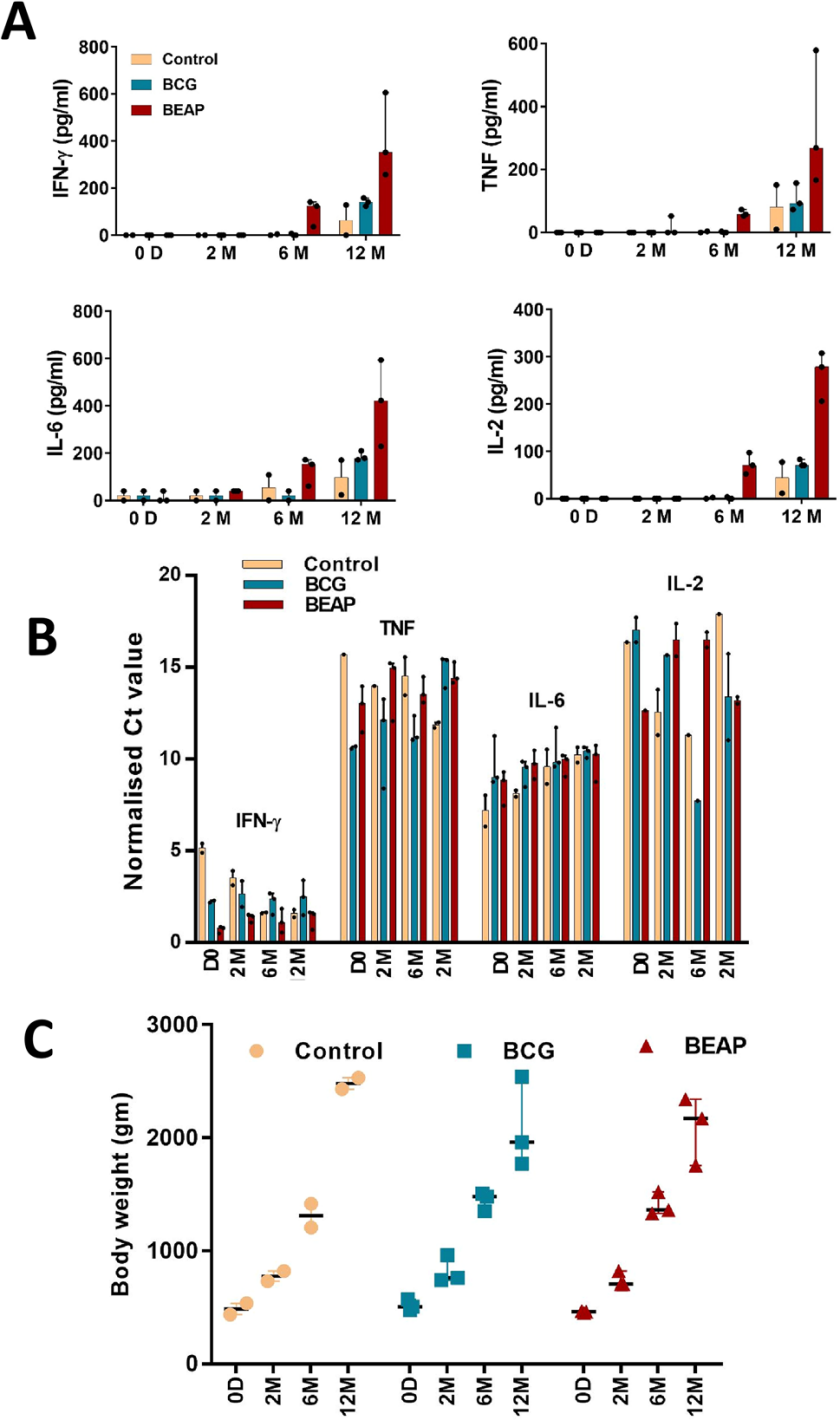
The non-toxic response of BCG/BEAP. (**A**) Cytokines levels, estimated by the cytometric bead array (BD Biosciences), in serum post immunization. (**B**) The m-RNA levels of the cytokine genes after normalization with the housekeeping gene GAPDH. (**C**) The progressive growth in terms of body weight of immunized and control animals up to 12 months. Dots indicate data points and bars are median with 95% confidence interval. Graphs were plotted using GraphPad Prism version-7 program (https://www.graphpad.com/).

Cytokines which are typically associated with inflammation like TNF, IL-6, IFN-γ and IL-2 were checked in serum and are shown in Fig. 2A. While only IL-6 was detectable on Day 0, all 4 cytokines showed a gradual increase in levels only after 2 months and detectable on 6 months and 12 months post immunization. The cytokine titres were generally higher in the BEAP immunized group. The m-RNA expression of these cytokine genes in the blood was also checked and is shown in Fig. 2B.

#### *CD4* + *and CD8* + *T cells count*

The CD4 + and CD8 + cells were found to be elevated, which also explains the elevated cytokine levels. However, no pattern with respect to age and different time points was apparent. Details are described in the Supplementary data, Figure S1 and S2.

#### Growth of immunized macaque infants

The bodyweight of the control and the BCG/BEAP immunized infants were measured on the days of blood collection. It was observed that the total body weight of infants was progressive with no symptoms of weakness or weight loss in the immunized animals. The median body weight in grams of all infants on day 0, 2 months, 6 months, and 12 months were 470 interquartile range (IQR) 55 gm, 750 IQR 96 gm, 1387 IQR 141 gm and 2255 IQR 542 gm respectively (Fig. 2C).

#### Chest radiography

The postero-anterior chest X-ray of the control and immunized infants was done after 12 months of immunization. Both the lung fields and chest were clear in all the X-ray images. No abnormalities were seen.

#### Behaviour monitoring

The behaviour of the infants was observed after the immunization. It was observed that most of the time the infants were in contact with their mother. Proper milk feeding and food provided externally were also consumed by the infant suggesting no difficulty in swallowing. Moreover, no sign of any external infection or bruises was observed.

### Assessment of the efficacy of BEAP

#### Stimulation of T cells with M.tb antigen

In order to compare the protective efficacy, lymphocytes isolated from blood, primarily comprising of T cells, were stimulated with *M.tb* antigen for 8 days and levels of INF-γ, TNF, IL-6 and IL-2 were estimated in the culture supernatant.

**Figure 3:**
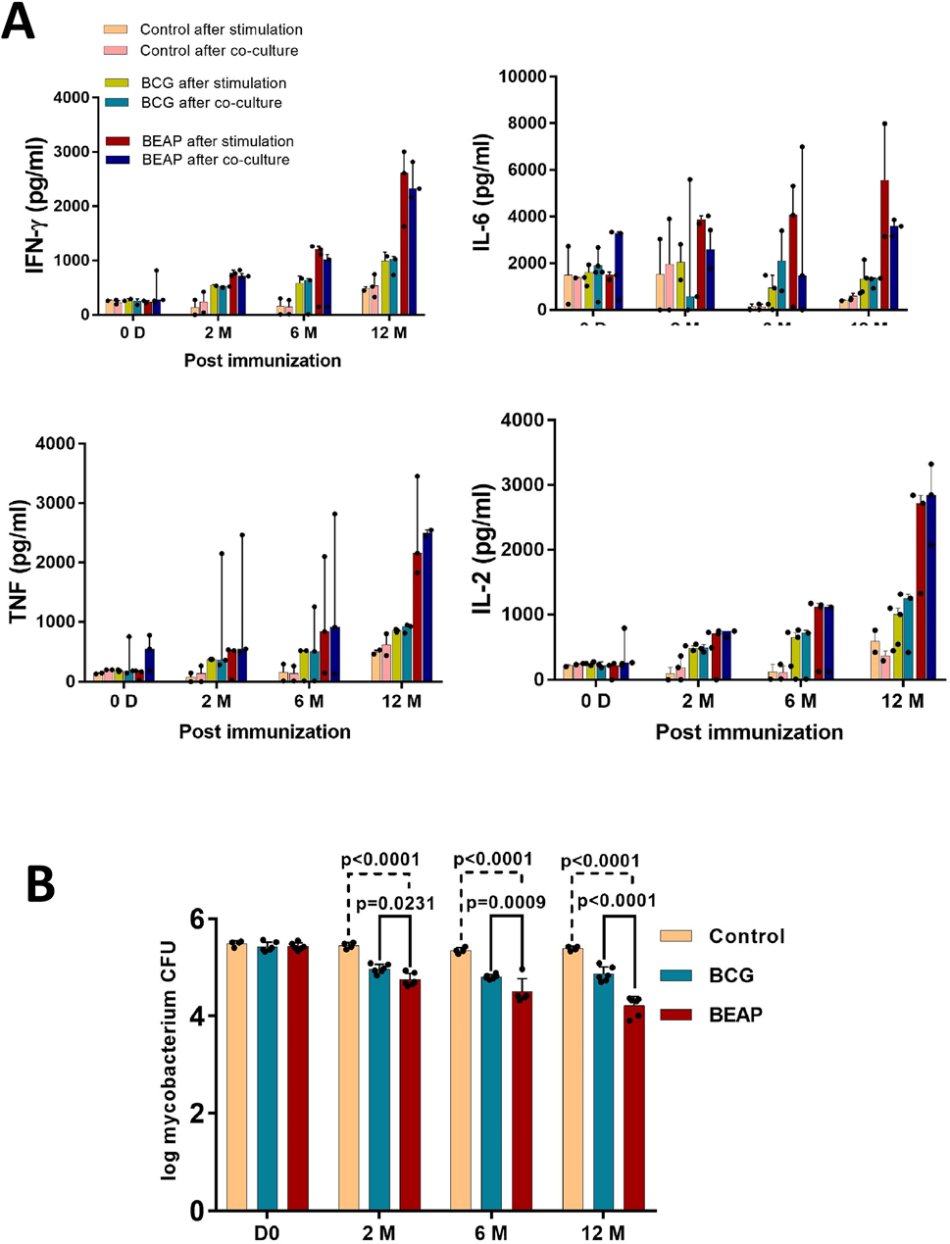
Efficacy and immune response elicited by BCG/BEAP. (**A**) The cytokine secretion profile in the supernatant of *M.tb* antigen stimulated T cells and when activated T cells were co-cultured with *M.tb* infected macrophages from the respective animals. Dots indicate data points and bars are median with 95% confidence interval. (**B**) Mycobacterium colony-forming units obtained after *M.tb* infected macrophages and activated T cells were co-cultured. The data demonstrate a substantial decrease in the mycobacterium CFU in the BEAP and BCG immunization compared to control animals. Dots indicate CFU counts calculated from multiple plates and bars are mean ± SD and analysis was done using two-way ANOVA with Tukey’s correction for multiple comparisons. Graphs were plotted using GraphPad Prism version-7 program (https://www.graphpad.com/).

The difference in cytokine levels between immunized and control groups became more pronounced after 6 months of immunization and at 12 months the levels were the highest in the BEAP group (Fig. 3A), suggesting that in the BEAP immunized group there was enhanced activation of T cells.

#### Co-culture of activated T-cells and M.tb infected macrophages

*M.tb* antigen stimulated effector T cells, when co-cultured with *M.tb* infected macrophages, generate inflammatory cytokines that enhance the activity of macrophages to eliminate the mycobacteria. As observed in the T cells stimulation by *M.tb* antigen experiment, in the co-culture experiment too, low levels of cytokines were detected in the control group up to 12 months. Among the BEAP and BCG groups, higher secretion of cytokines was observed in the BEAP group.

After the co-culture, a 1.2 log decrease in the mycobacterium CFU was observed in the BEAP immunized group compared to the BCG group (0.56 log) between 0 day and 12 months (Fig. 3B). Overall, BEAP showed lowest number of mycobacteria CFU followed by the conventional intradermal BCG vaccination.

## Discussion

In this study we investigated the safety and efficacy of a new BCG formulation, BEAP, which was delivered intra-tracheally in infant macaques and explored how BEAP could be compared with the conventional intradermal BCG immunization.

Before performing the intratracheal intubation and delivery of the DPA of BEAP in infants, the procedure was evaluated on an adult *macaque*. The protocol was well tolerated by the adult animal; the whole procedure was completed within a couple of minutes and no adverse reaction was observed for up to two months.

The immunization of infant *macaque* by BCG or BEAP did not cause an adverse reaction as there were no significant changes observed in the WBC and granulocytes counts post-immunization. Post immunization, the normal growth and behaviour of the animals, clear chest radiography and normal levels^14^ of liver secreted enzymes indicated the non-toxicity of BCG and BEAP in the *macaque* model.

The immunized animals had higher levels of INF-γ, TNF, IL-6 and IL-2 in the serum especially in the BEAP immunized group. Further, the genes for these cytokines were expressed in the WBCs (Fig. 2B) even though these cytokines were not detected in serum at 0 to 2 months’ time points after immunization. The increased cytokine levels did not show any adverse effect on the health of the macaques.

So far there is no reported ‘normal range’ of these cytokines in the serum after the BCG immunization in *macaque* infant. It is likely that the elevated but tolerable higher inflammatory condition caused by TNF and IL-6 post BCG or BEAP immunization would provide ‘non-specific’ innate immune protection against environmental antigens. On the other hand, higher levels of INF-γ and IL-2 post BCG immunization in children is a typically observed phenomena^15^ and it correlates with the degree of protection against *M.tb* infection^16^. Concurrently, we observed higher levels of IL-2 and INF-γ in BCG and BEAP immunized animals indicating the non-specific protection often attributed to BCG immunization^17,18^. The elevated cytokines indicate vaccine mediated prophylaxis which is imperative for innate immunity activation and subsequent generation of adaptive immunity^15,19^.

Upon stimulation of T cells with *M.tb* antigen, the resulting cytokine milieu leads to the proliferation of effector T cells which can effectively eliminate *M.tb* infected macrophages^20,21^. Such a response has been considered as an indication of the BCG vaccine generating a protective response^22,23^.

Our data suggests that upon immunization, animals would be able to generate optimal effector T cell response, wherein IL-2 mediates effector T cell proliferation, IL-6 mediates survival and INF-γ along with IL-6 mediate pleiotropic effect for T cell lineage shift to Th1 cells^24–26^. Similar findings have been made by Min et al^27^, where healthy and naturally *M.tb* infected rhesus monkey’s whole blood was stimulated with a purified protein derivative (PPD). They identified, IL-2, IL-6 and INF-γ among a few others as potential biomarker for tuberculosis.

It was observed that the effector T cells in BEAP and BCG immunized groups were efficient in eliminating *M.tb* compared to the control group (Fig. 3B). This correlates well with the elevated levels of inflammatory cytokines upon stimulation of T cells with *M.tb* antigen and co-culture of effector T cells with *M.tb* infected macrophages.

This assay is typically known as the mycobacterium growth inhibition assay (MGIA). Many reports advocate the advantages of this assay in vaccine testing against TB^28–32^ and this has enabled monitoring the immunological development in the same animals over time.

The aerosol immunization with BCG had raised some attention in the past^33^ and there have been a few attempts to evaluate the aerosol immunization with BCG in human subjects^34^. Barclay et al^35^ demonstrated higher degree of protection against TB infection in macaques after aerosol immunization with BCG. This was taken up more systematically by White et al.^36^ and they evaluated the immunogenicity of BCG delivered by the aerosol to the lungs of macaques. They examined the immune response post immunization in detail and demonstrated that after immunization there was an increase in the frequencies of IFN-γ, TNF-α and IL-2 producing cells in the peripheral blood mononuclear cells (PBMCs) and bronchoalveolar lavage (BAL) fluid. The trends of cytokines levels post immunization and overall conclusions of this study corroborate well with earlier findings.

While interpreting the efficacy of BEAP, it is important to be aware that the modulation of cytokine levels with age, post immunization or upon stimulation with antigens, is a complex phenomenon^37^ and cannot be linked directly to the immunogenicity or efficacy of a vaccine.

It is of considerable interest to compare immunization by the DPA of BEAP with a few alternative BCG candidate vaccines and delivery strategies.

MVA85A is a modified Vaccinia Ankara virus expressing antigen-85A candidate vaccine designed to boost BCG induced immunity^38^. This vaccine when delivered by the aerosol route was found to be safe and highly immunogenic in macaques^39^. Aerosol and intradermal booster immunization by MVA85A among BCG immunized people are in the advance stage of clinical trials^40,41^.

The intravenous (IV) injection of BCG has gained some attention recently but the IV injection of BCG to TB patients has been reported before the invention of antibiotics therapy^42^ and it is generally accepted that the IV administration of BCG provides enhanced protection against *M.tb* infection^43^. This has been confirmed by several investigators; Sharpe et al^44^ demonstrated that following intravenous immunization by BCG, the *M.tb* infection resulted in reduced disease pathology due to IFN-γ and TNF-α producing CD4 T cells. However, high frequencies of this cell population lead to increased pathology. More recently the role of systemic and tissue resident T cells after intravenous BCG immunization has also been emphasized^45^.

While investigating the intravenous routes, so far the major emphasis has been to achieve higher efficacy against *M.tb* infection and typically a high dose of BCG (around 10^7^ CFUs) is investigated and recommended^45^. The safety aspect of such a dose is yet to be fully established as the higher frequency of IFN-γ producing CD4 + cells are also known to be responsible for pathogenesis and Tuberculosis-immune reconstitution inflammatory syndrome (TB-IRIS)^46^.

On the other hand, for the BEAP there are fewer safety concerns as only very few, 1000–1500 BCG bacilli are delivered; this is even fewer than conventional intradermal BCG immunization in which typically 5 × 10^5^ – 5 × 10^7^ BCG bacilli are administered. Moreover, the BEAP DPA delivery system will be free of the needle and a syringe.

Overall, our findings indicate that the BCG encapsulated alginate particle (BEAP) is a promising candidate vaccine for TB among a few others^47–50^. It would be of interest to investigate how without any genetic modification in the BCG, the BEAP presented enhanced protection. In most likelihood, it is a combination of several factors like the route of administration, alginate encapsulation of BCG which leads to efficient phagocytosis and activation of lung DCs^12^, size of particles etc., apart from the mechanism of the limited protection provided by the BCG itself which is not yet fully understood^51^.

This study confirms that when the BEAP is delivered as DPA to infant *Rhesus macaques*; it is likely to be non-toxic, does not generate a hypersensitive response and elicits an immune response which may provide protection from the TB infection. The ease of delivery and the longer shelf life of at least six months at room temperature are the additional advantages of BEAP^12^.

### Limitation of the study

This pilot study gives encouraging leads to further elaborate the investigation. Since the number of subjects in each group is minimal (2 un-immunized controls, 3 BEAP immunized and 3 BCG immunized macaque infants), this needs to be further extended on more non-human subjects to make statistically significant comparisons of findings among different groups.

Secondly, the TC-MGIA findings are suggestive of expected outcomes; however, confirmation by *in-vivo* infection would add significantly to establish the pre-clinical safety and efficacy of BEAP vaccine formulation.

## Methods

### Preparation of BCG encapsulated alginate particle

The BEAP formulation was prepared and characterized as described in Nagpal et al. 2019^12^. Briefly, 1 × 10^10^ live BCG bacilli was mixed in 50 ml solution of sodium alginate (Fluka, Catalog number: 71238) (1.23%) and trehalose (Sigma Aldrich, Catalog Number: 90210) (8.25%) solution. The mixture was filled in a nebulization assembly and aerosol was generated by a piston-based air pump. The generated aerosol was passed through a 5% calcium chloride solution resulting in the encapsulation of aerosol droplets (HiMedia, Catalog number: GRM3906). As a result, a gel-like solution was formed. It was centrifuged at 350 g for 10 min and washed three times with distilled water to completely remove the remaining calcium chloride. The final pellet was re-suspended in 5 ml distilled water and lyophilized. The lyophilized product was further processed through a jet mill (Sturtevant, USA) to obtain an inhalable powder of 3–5 µm particle size. The BEAP formulation was then characterized for its size, stability, viability and release profile of BCG.

### Experimental animals and husbandry

The study was approved by the Institutional Animal Ethics Committee of the National Institute of Immunology (NII) file number (IAEC#316/13). All the methods were performed in accordance with the relevant guidelines and regulations under the supervision of a professional Veterinarian at the primate research facility of NII, New Delhi. All animal experiments and reporting adhere to the ARRIVE guidelines^52^.

Experiments were performed on two adults and eight infant macaques. One of the adult animals was immunized intradermally using a conventional BCG vaccine and the other was immunized intratracheally with DPA of BEAP. Three infants each were immunized either with BEAP intratracheally or with BCG intradermally. There were two control or unimmunized infants.

The infant mainly feeds on adult mother milk. Lactating mothers were fed with an adequately nutritious diet every day which comprises of soaked gram seed (75–100 gm), bread slices (4–5 slices), dry pellet diet (100 gm), and fresh vegetables/fruits (500–600 gm); vitamins/calcium supplements were given alternatively five times a week. The drinking water was supplied ad libitum to the individual cages. Adult animals were fed similarly except bread slices (3–4 slices), fresh vegetables/fruits (450–500 gm); vitamins/calcium supplements were given alternatively twice in a week. The infants were kept with their mother in temperature-controlled rooms maintained in proper hygiene. For all the procedures, animals were sedated by intramuscular injection with ketamine hydrochloride (Ketajex 50 mg/ml, Claris injectable limited, India) at a dose of 5–10 mg per kg of body weight depending on the experimental needs. None of the animals had previously been used for any other experimental procedures. Before immunization, animals were weighed and physically examined for any gross abnormalities and were monitored for behavioural and clinical changes post immunization.

### Immunization

BCG immunization: Two-week old infants or adults were immunized via the intradermal route on the thoracic dorsal area with a single dose of freeze-dried live attenuated BCG Danish strain 1331 vaccine (B.No. 109006; GreenSignal, BioPharma Pvt Ltd, India). The lyophilized vaccine was re-constituted with the diluent provided by the manufacturer and 0.1 ml of the suspension was administered intradermally. The site was monitored for any local reaction after the vaccination.

BEAP immunization: Two-week-old Rhesus macaque infants or adults were immunized via the intratracheal route. The animals were anesthetized using ketamine hydrochloride injection. The animal was rested on a horizontal clean surface after ensuring the animal is fully anesthetized. The mouth of the infant was opened and its tongue was gently pulled out to visualise the epiglottis and tracheal opening of the infant. The salivary secretions were cleaned with sterile cotton gauzes. The intubation was guided and performed by using an otoscope. A sterile Poly vinyl chloride (PVC) tube of outer diameter 1.60 mm and an inner diameter of 0.80 mm was inserted into the trachea of the animal. The tracheal intubation was confirmed by placing a mirror at the outer end of the tube which became foggy due to moisture of warmer exhaled air. The BEAP formulation was administered through the tube into the lung of the animal by a 1 ml syringe using an in-house designed aerosol delivery apparatus^12^. Briefly, the aerosol of BEAP was generated and filled in a 10 ml syringe. By using a three-way valve, 1 ml of air saturated with BEAP aerosol was withdrawn from the 10 ml aerosol filled syringe and delivered into the infant’s lung.

This procedure was repeated 3 times followed by 3 additional pushes of clean air. Typically, the amount of BEAP transferred contained 1000–1500 BCG CFU. After the delivery of BEAP, the animal was laid on a flat surface until the animal recovered from anaesthesia and subsequently housed with the mother.

### Blood and serum collection

The blood was collected from the animals in citrate phosphate dextrose (CPD) anticoagulant containing syringe at zero-day (day of immunization), 2 months, 6 months, and 12 months post immunization. During the standardization of immunization procedures performed on adults, animal’s blood was collected only on the day of immunization and at 2 months post immunization. For serum isolation blood was collected in a vial without CPD. The serum was isolated by spinning the coagulated blood at 12,000 g for 10 min at 4 °C and stored at -80 °C until further analysis.

### Cytokine analysis

The cytometric bead array (CBA) (BD Non-Human Primate (NHP) Th1/Th2 Cytokine Kit (Catalog No. 557800) was used to quantify the cytokine levels in the serum and the culture supernatant. The assay was performed as per the manufacturer’s protocol. Briefly, 10 µl of capture beads for each cytokine (IFN-γ, TNF, IL-6, IL-2) per analyte were mixed to prepare a cocktail of capture beads. Similarly, a cocktail of detection antibody for each cytokine was also prepared. All the standards provided with the kit for each cytokine, were mixed in 2 ml assay diluent and incubated at RT for 15 min. Next, in each analyte tube 60 µl of capture beads cocktail and 60 µl of sample/standard, was mixed. Finally, 60 µl of PE detection antibody was added. The mixture was incubated at RT for 3 h in dark. In the last step, the mixture was washed with 1000 µl wash buffer and re-suspended in 300 µl wash buffer. The stained sample was run in the flow cytometer BD FACSCanto. The results were analysed using FCAP array V3 software(https://www.bdbiosciences.com/us/applications/research/bead-based-immunoassays/analysis-software/fcap-array-software-v30/p/652099#).

### RNA isolation

RNA was isolated from the WBCs of the infants using the MasterPure Complete DNA and RNA Purification kit (Lucigen, Catalog Number: MC85200) as per the manufacturer’s instruction. The RNA pellet obtained was resuspended in TE buffer and quantified on a nano-drop machine. One µg of RNA was used to synthesize cDNA.

### cDNA synthesis and qRT-PCR

RT-PCR was performed to quantitate the mRNA expression level of different cytokine genes. For this cDNA was synthesized from the 1 µg RNA using an iScript cDNA Synthesis kit (Bio-Rad, Catalog number: 1708891) as per the manufacturer’s instructions. The expression of the cytokine genes was confirmed by quantitative PCR using GeneSure SYBR Green qPCR Master Mix (Puregene, Catalog number: PGK025-A). The following PCR program was used: initial denaturation for 10 min (single step) at 95 °C, followed by 40 cycles of, denaturation at 95 °C for 15 s, annealing between 60.2 °C and 65 °C for 15 s, extension at 72 °C for 15 s followed by a single-step melting curve. The primers for different cytokine genes having specificity to Rhesus were designed by Beacon Designer 7.7 software (http://www.premierbiosoft.com/molecular_beacons/). The sequence of the primers is given in Supplementary data Table 1.

### T-cell based Mycobacterium growth inhibition assay (TC-MGIA)

The TC-MGIA is an advanced technique similar to PBMC-MGIA used to study vaccine-mediated host immune response *ex-vivo* and was employed to analyse the efficacy of BCG and BEAP formulation in infants. The assay was performed according to Worku and Hoft^20^ with some modifications. Briefly, 2 ml of blood from the immunized or control infant was collected in syringes containing CPD anticoagulant. The PBMC was isolated from the blood using HiSep LSM 1077 (HiMedia, Catalog number: LS001) based density gradient centrifugation. Cells from the buffy layer were collected and washed with phosphate buffer saline (PBS) (HiMedia, Catalog number: TS1006) and plated in a TC-25 flasks (Corning, Catalog number: 430639) for 3 h to allow the monocytes to adhere. The non-adherent cells which contain the T cell, was collected and were stimulated with *M.tb* H37Rv antigen (2 µg/ml) in IMDM media (HiMedia, Catalog number: AL070) containing 10% FBS (Gibco, Catalog number: 10270), 1% antibiotics and anti mycotic cocktail (HiMedia, Catalog number: A0021) for 8 days. The adherent monocytes were harvested by trypsinization and seeded on collagen-coated 96 well plates (Corning, Catalog number: 3599) (0.13 × 106 cells per well) in IMDM media) containing 10% FBS, 1% antibiotics and anti mycotic cocktail, and 30 ng MCSF growth factor (Prospec, Catalog number: CYT-308). Cells were incubated at 37 °C, 5% CO_2_, and 85–90% humidity for 6 days to differentiate into macrophages. After 6 days, the macrophages were infected with *M.tb* H37Rv with a multiplicity of infection (MOI) of 1:10 for 6 h. Cells were then washed and incubated for 48 h and co-cultured with *M.tb* antigen-stimulated T cell for 48 h. Finally, the mycobacterium growth inhibition was checked by lysing the infected macrophages with 0.2% saponin and plating on Middlebrook 7H11 agar plates. Plating was done in triplicate and at least at 3 log dilutions. Plates were incubated at 37 °C for 2–3 weeks and the resulting colonies were counted.

### Haematology, LFT and chest X-ray

The haematology and Liver function tests were performed after immunization BCG/BEAP vaccine. Haematology was performed with fresh blood using Haematology Analyzer MS 4e Automated Cell Counter. The LFT and cholesterol were checked in the serum using respective enzymatic assay kits (Coral Clinical system, India) as per the manufacturer’s protocol. A postero-anterior (PA) chest X-ray was performed in-house.

### Statistical analysis

All the data were statistically analysed using GraphPad Prism version-7 program (https://www.graphpad.com/). Data was plotted as median with 95% confidence interval, except Fig. 3B in which mean ± standard deviation (SD) was plotted and the ANOVA variance test using Turkey’s correction was made among the groups.

## Supplementary Information

Supplementary Information.

## Data Availability

All data generated or analyzed during this study are included in the manuscript and its supplementary data.
