# Supplementary material for: The safety and efficacy of BCG encapsulated alginate particle (BEAP) against M.tb H37Rv infection in Macaca mulatta : A pilot study
Source: Sci Rep. 2021 Feb 4;11:3049. doi: 10.1038/s41598-021-82614-5 (PMC7862294; doi:10.1038/s41598-021-82614-5)
Supplement: Supplementary file 1 — Supplementary Information. [file 41598_2021_82614_MOESM1_ESM.docx]

**The safety and efficacy of** **BCG encapsulated alginate particle (BEAP) against** ***M.tb* H37Rv infection in *Macaca mulatta* : A pilot study**

Ashwani Kesarwani^1,2^, ashwani.kesar@gmail.com

Parul Sahu^1^, parulsahu18@nii.ac.in

Kshama Jain^1^, jainkshama@nii.ac.in

Prakriti Sinha^1^, prakriti@nii.ac.in

K Varsha Mohan^1^, kvarshamohan@nii.ac.in

Puja S Nagpal^1^, pujanii@gmail.com

Surender Singh^1^, surenders@nii.ac.in

Rana Zaidi^2^, rzaidi@jamiahamdard.ac.in

Perumal Nagarajan^1^, nagarajan@nii.ac.in

Pramod Upadhyay^1^, pkumar@nii.ac.in

1 National Institute of Immunology, Aruna Asaf Ali Marg, New Delhi 110067

2 Department of Biochemistry, Jamia Hamdard, New Delhi, 110062

**Contents**

**Item Page**

Table 1: List of Primers used in the study 3

Table 2 : Complete blood count (CBC) parameters for immunized and control animals at different time points 4

Method for CD4+ and CD8+ T cells count (Figure S1) 7

Results of CD4+ and CD8+ T cells count (Figure S2) 8

**Table 1: List of Primers used in the study.**

| **S.No** | **Gene Name** | **Forward**  **5’-3’** | **Reverse**  **5’-3’** |
| --- | --- | --- | --- |
| **1.** | **IFN-γ** | CGAATGTCCAACGCAAAGCA | GGGATGCTCTTCGACCTCGA |
| **2.** | **TNF** | GGCTCAGGCAGTCAGATCA | GCTTGAGGGTTTGCTACAACAT |
| **3.** | **IL-6** | GAGTAGTGAGGAGCAAGCCA | CATTTGTGGTTGGTTCAGGGG |
| **4.** | **IL-2** | ATCCCAAACTCACCAGGATGC | AGATGTTTCAATTCTGTGGCCTTC |
| **5.** | **GAPDH** | GGAAGGTGAAGGTCGGAGTC | GGCAACAATATCCACTTTA |

**Table 2 : Complete blood count (CBC) parameters for immunized and control animals at different time points.**

**Animals immunized with BEAP,** *(*) indicate missing data point*

|  |  |  | **BEAP 1** | | | | **BEAP 2** | | | | **BEAP 3** | | | |
| --- | --- | --- | --- | --- | --- | --- | --- | --- | --- | --- | --- | --- | --- | --- |
| **Parameters** | **Range** | **Units** | **D0** | **2M** | **6M** | **12M** | **D0** | **2M** | **6M** | **12M** | **D0** | **2M** | **6M** | **12M** |
| **MONOCYTES** | 0-0.9 | m/mm^3^ | 0.59 | 0.82 | 0.84 | 0.89 | 0.86 | 0.89 | 1 | 0.1 | 0.41 | 0.22 | 0.53 | 0.89 |
| **GRANULOCYTES** | 1.4-13.2 | m/mm^3^ | 0.39 | 0.51 | 0.12 | 0.42 | 0.26 | 2.37 | * | 2.36 | 3.05 | 3.83 | 4.72 | 3.41 |
| **RBC** | 4.1-5.7 | M/mm^3^ | 3.55 | 5.51 | 4.52 | 5.39 | 4.9 | 4.22 | 2.93 | 3.48 | 3.46 | 3.48 | 4.2 | 4.73 |
| **MCV** | 71.0-103.0 | fl | 105.9 | 103 | 97.4 | 91.4 | 108.4 | 88.4 | 85.3 | 92.4 | 93.2 | 87.3 | 91.2 | 90.7 |
| **HCT** | 29.6-55.3 | % | 37.5 | 56.7 | 44 | 49.2 | 53.1 | 44.3 | 25 | 32.1 | 32.2 | 30.3 | 38.3 | 42.9 |
| **MCH** | 16-24 | pg | 32.4 | 27.9 | 24.5 | 24.3 | 26.1 | 21 | 30 | 26.4 | 27.7 | 25.8 | 25 | 23.8 |
| **MCHC** | 28-36 | g/dl | 30.9 | 27.1 | 25.2 | 26.6 | 24.1 | 31 | 35.2 | 28.6 | 29.8 | 29.7 | 27.4 | 26.3 |
| **RDW** | 4.0-7.0 |  | 9.8 | 9.8 | 9.8 | 9.5 | 10.6 | 6.5 | * | 9.7 | 9.1 | 9 | 9.9 | 9.5 |
| **Hb** | 9.5-16.3 | g/dl | 11.6 | 15.4 | 11.1 | 13.1 | 12.8 | 10.2 | 8.8 | 9.2 | 9.6 | 9 | 10.5 | 11.3 |
| **THR** | 72-519 | m/mm^3^ | 679 | 763 | 366 | 365 | 820 | 444 | 406 | 169 | 222 | 183 | 223 | 254 |
| **MPV** | 4.0-7.0 | fl | 8.2 | 8.1 | 7.9 | 8.3 | 8 | 3.9 | * | 8.2 | 8 | 7.9 | 7.5 | 7.7 |
| **Pct** |  | % | 0.56 | 0.62 | 0.29 | 0.3 | 0.66 | 0.22 | * | 0.14 | 0.18 | 0.14 | 0.17 | 0.2 |
| **PDW** | 4.0-7.0 |  | 8.5 | 8.1 | 7.5 | 9 | 8.4 | 4.6 | * | 7.2 | 8 | 8.2 | 7.1 | 6.5 |

***Table continues to next page :***

***Table continues from previos page :***

**Animals immunized with BCG,** *(*) indicate missing data point*

|  |  |  | **BCG 1** | | | | **BCG 2** | | | | **BCG 3** | | | |
| --- | --- | --- | --- | --- | --- | --- | --- | --- | --- | --- | --- | --- | --- | --- |
| **Parameters** | **Range** | **Units** | **D0** | **2M** | **6M** | **12M** | **D0** | **2M** | **6M** | **12M** | **D0** | **2M** | **6M** | **12M** |
| **MONOCYTES** | 0-0.9 | m/mm^3^ | * | 0.55 | 1.27 | 0.1 | 0.48 | 1.44 | 2.18 | 0.11 | 0.33 | 0.16 | 1.21 | 0.63 |
| **GRANULOCYTES** | 1.4-13.2 | m/mm^3^ | * | 0.16 | 0.18 | 2.35 | 0.06 | 1.33 | 0.34 | 3.03 | 1.55 | 2.21 | 8.55 | 2.2 |
| **RBC** | 4.1-5.7 | M/mm^3^ | * | 4.51 | 3.05 | 3.39 | 6.38 | 4.99 | 5.74 | 3.51 | 3.51 | 3.21 | 4.14 | 4.31 |
| **MCV** | 71.0-103.0 | fl | * | 103.9 | 108 | 94.8 | 104 | 98.66 | 95.3 | 92.1 | 101.3 | 81.1 | 96.1 | 91.8 |
| **HCT** | 29.6-55.3 | % | * | 46.9 | 33.1 | 32.1 | 66.5 | 49.2 | 54.7 | 32.3 | 35.5 | 26 | 39.7 | 39.5 |
| **MCH** | 16-24 | pg | * | 23.2 | 23.9 | 25.9 | 28.2 | 23.4 | 25.4 | 26.4 | 31.3 | 22.7 | 26 | 24.5 |
| **MCHC** | 28-36 | g/dl | * | 22.4 | 22 | 27.4 | 27.2 | 23.7 | 26.6 | 28.7 | 30.9 | 28 | 27.2 | 26.8 |
| **RDW** | 4.0-7.0 |  | * | 11.1 | 11.7 | 9.2 | 9.8 | 10.3 | 9.1 | 9.2 | 10.4 | 9 | 9.8 | 10.5 |
| **Hb** | 9.5-16.3 | g/dl | * | 10.5 | 7.3 | 8.8 | 18.1 | 11.7 | 14.6 | 9.3 | 11 | 7.3 | 10.8 | 10.6 |
| **THR** | 72-519 | m/mm^3^ | * | 944 | 588 | 149 | 73 | 580 | 393 | 213 | 94 | 175 | 300 | 157 |
| **MPV** | 4.0-7.0 | fl | * | 8.1 | 8.3 | 8.6 | 8.2 | 7.9 | 8 | 7.8 | 8.4 | 7.4 | 7.9 | 7.8 |
| **Pct** |  | % | * | 0.76 | 0.49 | 0.13 | 0.06 | 0.46 | 0.31 | 0.17 | 0.08 | 0.13 | 0.24 | 0.12 |
| **PDW** | 4.0-7.0 |  | * | 8.5 | 8 | 7.6 | 11 | 7.6 | 8.8 | 6.3 | 8.2 | 6.8 | 6.4 | 8.6 |

***Table continues to next page :***

***Table continues from previos page :***

**Control unimmunized animals**

|  |  |  | **CONTROL 1** | | | | **CONTROL 2** | | | |
| --- | --- | --- | --- | --- | --- | --- | --- | --- | --- | --- |
| **Parameters** | **Range** | **Units** | **D0** | **2M** | **6M** | **12M** | **D0** | **2M** | **6M** | **12M** |
| **MONOCYTES** | 0-0.9 | m/mm^3^ | 1.06 | 0.57 | 1.25 | 0.95 | 0.44 | 0.88 | 1.51 | 0.44 |
| **GRANULOCYTES** | 1.4-13.2 | m/mm^3^ | 10.72 | 3.84 | 8.86 | 4.41 | 3.09 | 5.03 | 9.37 | 1.92 |
| **RBC** | 4.1-5.7 | M/mm^3^ | 2.92 | 2.77 | 4.64 | 4.49 | 4.16 | 4.33 | 3.93 | 4.44 |
| **MCV** | 71.0-103.0 | fl | 92.7 | 90.5 | 87.8 | 82.8 | 92.3 | 92.7 | 93.4 | 88.4 |
| **HCT** | 29.6-55.3 | % | 27 | 25 | 40.7 | 37.1 | 38.3 | 40.1 | 36.7 | 39.2 |
| **MCH** | 16-24 | pg | 30.1 | 25.2 | 23.4 | 22.2 | 25.7 | 23.7 | 25.9 | 23.4 |
| **MCHC** | 28-36 | g/dl | 32.5 | 28 | 26.7 | 26.9 | 27.9 | 25.6 | 27.7 | 26.5 |
| **RDW** | 4.0-7.0 |  | 10.9 | 9.6 | 9.7 | 10 | 9.8 | 10.1 | 9.4 | 10 |
| **Hb** | 9.5-16.3 | g/dl | 8.8 | 7 | 10.9 | 10 | 10.7 | 10.3 | 10.2 | 10.4 |
| **THR** | 72-519 | m/mm^3^ | 187 | 191 | 232 | 188 | 52 | 244 | 261 | 185 |
| **MPV** | 4.0-7.0 | fl | 7.5 | 7.9 | 7.4 | 7.4 | 8.2 | 7.5 | 8.1 | 7.6 |
| **Pct** |  | % | 0.14 | 0.15 | 0.17 | 0.14 | 0.04 | 0.18 | 0.21 | 0.14 |
| **PDW** | 4.0-7.0 |  | 6.6 | 7.4 | 6.7 | 5.9 | 0 | 7.9 | 7.5 | 7.4 |

**Details on CD4+ and CD8+ T cells count**

**Method**

Blood was collected from Control, BCG and BEAP immunized animals in EDTA tubes and stained with anti-human CD3 (FITC) (BIOLEGEND, Catalog No. 300405), CD4 (PE) (BIOLEGEND, Catalog No. 301014) and CD8 (APC) (BIOLEGEND, Catalog No. 317410) antibodies. For staining, 50 μl antibody cocktail (1:200 dilution) was added to 50 µl blood and incubated at 4°C for 45 min, followed by RBC lysis and cell fixation with 1X BD Phosflow Lyse/Fix buffer (BD, 558049). The samples were incubated in lyse/fix buffer at 37°C for 15 min and further washed with 1X PBS twice and resuspended in 200 µl PBS and run on BD FACSVerse. Placed below (Figure S1) is a typical plot indicating gating strategy.


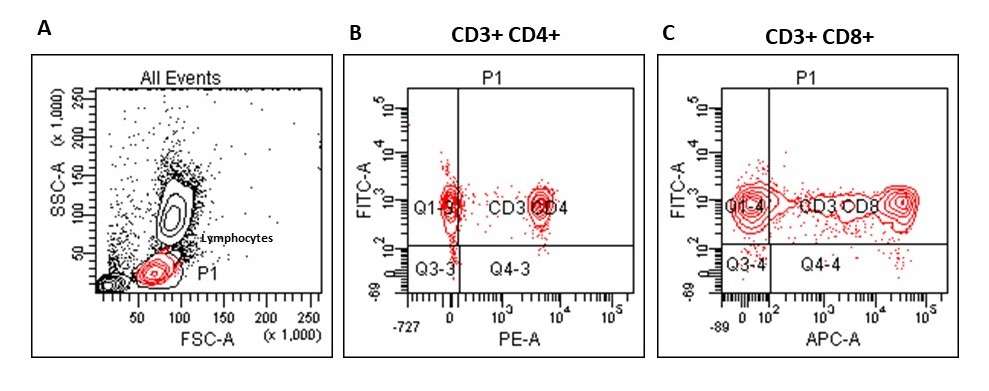


*Figure S1 : Representative graphs demonstrating the gating strategy used for calculating the percentage CD3+CD4+ and CD3+CD8+ population in whole blood. A: The SSC/FSC graphs showing the blood profile having gated lymphocytes as our cells of interest (P1). B: The Lymphocytes (P1) was further gated for double positive cells i.e. FITC-A (CD3) and PE-A (CD4). C: similarly, double positive cells FITC-A (CD3) and APC-A (CD8) were gated on P1 population. Appropriate single colour controls were also run for the compensation and the voltage settings.*

**Details on CD4+ and CD8+ T cells count**

**Results**

Typically, there was an increase in the CD4+ and CD8+ cell counts after two months of immunization in the BCG as well as the BEAP group but no specific pattern was observed at subsequent time points.
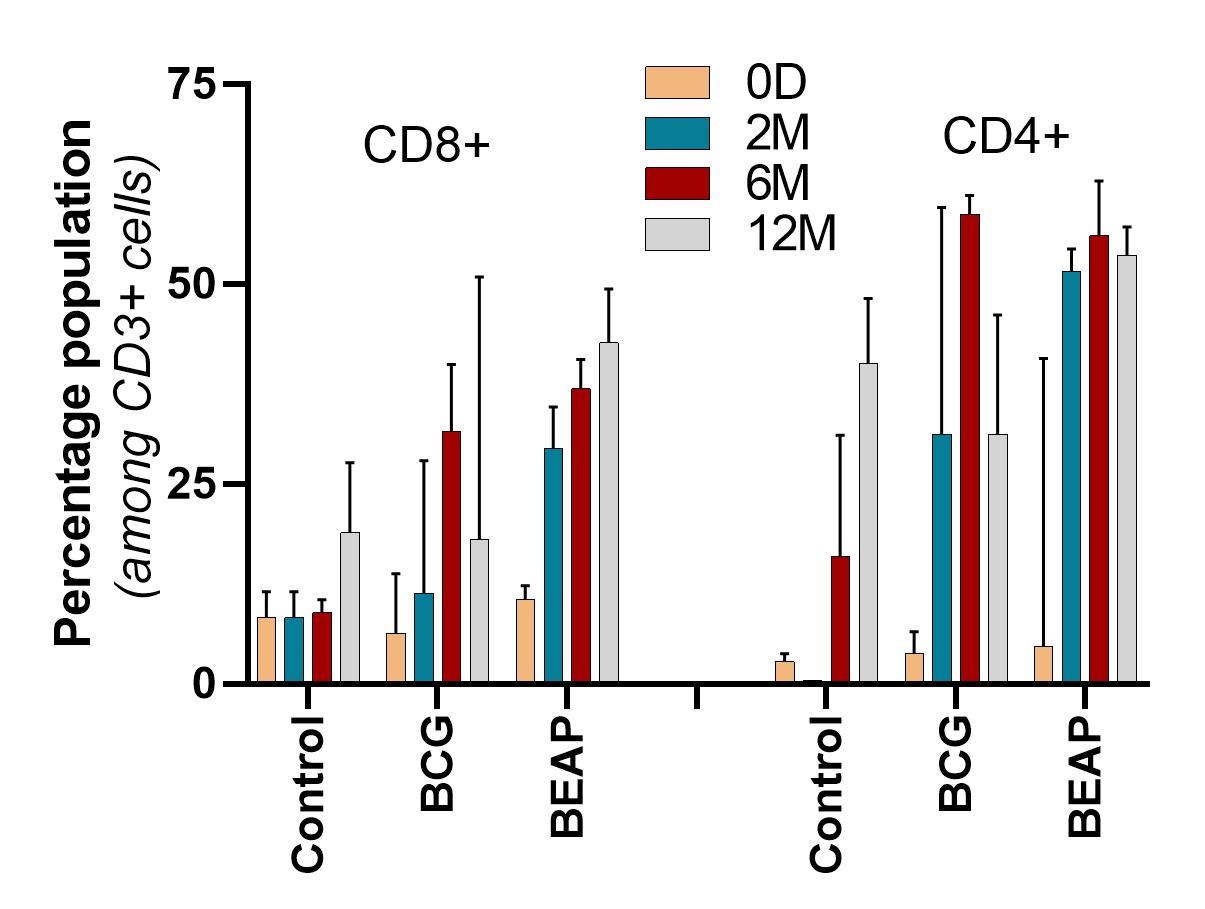


*Figure S2: Changes in the CD4+ and CD8+ population upon immunization at different time points. Graphs plotted using GraphPad Prism version-7 program (https://www.graphpad.com/).*
